# Exploring the Effect of Brief Preventive Videos on Mental Health Help‐Seeking for Early Psychosis in a Young Community Sample

**DOI:** 10.1111/eip.70007

**Published:** 2025-01-28

**Authors:** Clément Dondé, Louise Jambon, Marie‐Claire Wilhelm, Catherine Bortolon

**Affiliations:** ^1^ University Grenoble Alpes Grenoble France; ^2^ INSERM Grenoble France; ^3^ Psychiatry Department CHU Grenoble Alpes Grenoble France; ^4^ Psychiatry Department Centre Hospitalier Alpes‐Isère Saint‐Egrève France; ^5^ Grenoble INP, CERAG Grenoble France; ^6^ University. Grenoble Alpes, University Savoie Mont Blanc Grenoble France; ^7^ Institute Universitaire de Paris France; ^8^ Centre Référent Réhabilitation Psychosociale et Remédiation Cognitive (C3R), Centre Hospitalier Alpes Isère Grenoble France

**Keywords:** duration of untreated psychosis, early psychosis, prevention, stigmatisation, video

## Abstract

**Introduction:**

A key factor influencing the duration of untreated psychosis is that young individuals typically do not seek help during their initial psychotic experiences. This online study aimed to explore the efficacy of preventive video interventions providing information on psychosis on the attitudes towards seeking mental health care among young adults from the general population.

**Methods:**

Participants (*N* = 147) were randomised to one of the following online conditions: a short 3‐min video of an empowered patient or of a psychiatrist describing different aspects of mental illness, a short control video or no video. Then, participants answered the Inventory of Attitudes to Seeking Mental Health Services (IARSSM) to measure attitudes towards seeking mental health.

**Results:**

A Kruskal–Wallis one‐way ANOVA on the total IARSSM score revealed no significant effect of the group on attitude towards mental health care (χ2_(3)_ = 6.52, *p* = 0.09). A small but statistically significant effect was found for the IARSSM factor “indifference to stigma” (χ2_(3)_ = 8.50, *p* = 0.04), with slightly lower levels of indifference to stigma in the patient video group (*M* = 20.5, SD = 6.50) compared to the psychiatry video group (*M* = 24.5, SD = 4.35).

**Conclusion:**

Emphasising nonconformity with mental health stereotypes, portraying positive aspects and utilising short video formats on social media platforms can potentially reduce stigma in the short term. Long‐term effectiveness and identification of specific factors optimising attitudes towards mental health help‐seeking warrant further investigation.

## 1 Introduction

Psychotic disorders are significant health problems that typically start during the important development stages of adolescence and young adulthood. Duration of untreated psychosis (DUP) is defined as the time from manifestation of the first psychotic manifestations to initiation of adequate antipsychotic drug treatment. It averages around 1 year worldwide Salazar de Pablo et al. (2024). DUP is associated with more severe symptoms upon hospital or outpatient admission, reduced response to antipsychotic medications, elevated suicide risk, decreased social functioning and poor prognosis (Marshall et al. 2005; Penttila et al. 2014). To enhance the quality of life and outcome for youth with emerging psychosis, minimising DUP is crucial.

A critical factor influencing DUP is that young individuals typically do not seek help during their initial psychotic experiences (Martin et al. 2018). Less than one‐third (30%) of individuals in the early phase of psychosis seek professional psychiatric services (Addington, Van Mastrigt, and Addington 2004). Weaker intentions and behaviours in seeking help are associated with higher symptomatology, suggesting that those who need help the most are least likely to seek it (Aguirre Velasco et al. 2020). Stigmatisation was identified as the most significant barrier to seeking help for mental health in young people (Aguirre Velasco et al. 2020; Gulliver, Griffiths, and Christensen 2010), and recent systematic reviews emphasised its major role in hindering help‐seeking behaviours (Schnyder et al. 2017; Xu et al. 2018). Negative beliefs towards mental health services and professionals are other frequently cited obstacles to seeking help (Aguirre Velasco et al. 2020). A lack of information and awareness about mental illness symptoms and means to seek help also contributes to stigmatisation, leading to negative coping strategies like secrecy and avoidance, and eventually to delay in obtaining treatment (Hasan and Musleh 2017; Martin et al. 2018).

Seeking help for mental health issues involves communicating the need for personal and psychological assistance to obtain guidance and support. To do so, the individual must recognise the need for personal, psychological, emotional or social support, be willing to seek help and identify accessible sources of help, express symptoms and the need for assistance. A recently proposed conceptual framework for help‐seeking for mental health problems involves three intricate processes: attitudes, intentions and behaviours. Attitudes reflect the individual's perception of whether performing the behaviour would be a good or bad thing. Intentions reflect the plan or decision to seek help, which is determined by attitudes, subjective norms, that is, social stigma, and perceived behavioural control, that is, the individual's belief in the feasibility or ease of performing the behaviour. Behaviours are observable actions of seeking help. According to this framework, attitudes are a key and relevant target for improving help‐seeking intentions and behaviours (Rickwood and Thomas 2012).

Interventions to increase help‐seeking typically target attitudes by using strategies to improve mental health literacy, reduce stigma and promote motivational enhancement by increasing recognition of clinical manifestations, modifying dysfunctional beliefs about treatment and providing information about help sources (Xu et al. 2018). Social contact‐based video interventions have shown effectiveness in reducing stigma towards individuals with mental disorders and improving mental health literacy and help‐seeking attitudes in young people (Amsalem et al. 2021; Ito‐Jaeger et al. 2022; Janouskova et al. 2017; Morgan et al. 2018). However, video interventions to enhance help‐seeking are based on programmes mostly conducted in educational settings, and their effect in other community settings has been less investigated. Videos typically consist of clips of empowered patients describing different aspects of mental illness. The comparison of their effect with videos of recovery‐oriented mental health professionals discussing illness is unknown. More critically, less emphasis has been put on video interventions targeting specific disorders with long periods of illness without treatment, such as psychosis.

To address these knowledge gaps, this study aimed to explore the effect of preventive video interventions providing information on psychosis on the attitudes towards seeking mental health care among young adults aged 18 to 25 from the general population. In addition, we aim to compare the effects of a video of an empowered patient and a video of a recovery‐oriented psychiatrist on the attitude towards seeking care. Based on previous studies suggesting the effectiveness of patient videos with social contact in destigmatisation and help‐seeking behaviours (Amsalem et al. 2021; Morgan et al. 2018; Thornicroft, Mehta, and Clement 2016; Xu et al. 2018), we anticipate that groups exposed to preventive videos will show better attitudes towards seeking care than control groups, with the patient video group exhibiting the highest improvement. Two control conditions will be included. One with a video of a random man talking about a bike trip to Barcelona. The second control condition is a no‐video condition.

## 2 Methods

### 2.1 Participants

Assuming an effect size in ANOVA with fixed factors for groups (patient video, psychiatrist video, control video, no video) of Cohen's *d* = 0.258 based on results from a recent meta‐analysis (Morgan et al. 2018), the calculated sample size required to demonstrate a difference with 80% power (*p* = 0.05) was 130 participants. Considering a nonadherence rate of 10%–15% across all arms, the final sample size was determined to be 148 patients, that is, 37 per group (patient video, psychiatrist video, control video, no video). Subjects of both genders aged 18–25 years who were not working in the field of mental health were not included. Participants were recruited primarily through social media (e.g., Facebook, Twitter and Instagram) or flyers in various public locations in Grenoble (e.g., libraries, university restaurants, music schools, sports rooms and bakeries). The presented theme was ‘mental health of young adults’, and participants were invited to respond anonymously to a 10‐ to 15‐min online questionnaire via a provided link or QR code.

After completing the consent form, participants answered demographic questions related to inclusion criteria (age and job). Volunteers who were working in the field of mental health were excluded and redirected to the end of the study; otherwise, participants responded to five questions assessing their familiarity with the field of psychological assistance (Qualtrics) then randomly assigned one of the four conditions. After video viewing (if applicable), participants answered video control questions (see below). Then, participants responded to the 24 IARSSM questions, followed by questions measuring their familiarity with the field of mental health assistance. A preliminary study version was tested with eight volunteers for error checking, question comprehension and administration ease. Minor adjustments were made before implementing the final version. Descriptive and inferential statistical analyses, including Kruskal–Wallis and Spearman correlations for nonparametric data, were conducted using Jamovi version 2.3 (project 2024). The experiment was approved by the local research ethics committee (CERGA, University Grenoble Alpes, France).

### 2.2 Video Intervention

Each video lasted 3 min and 5 s and featured a 30‐ to 40‐year‐old man continuously speaking to the camera with subtitles. The development of the video (i.e., duration and male participant) was guided by practical and ethical reasons, including the fact that only a male patient agreed to disclose his personal experiences in the context of this study. The men introduced themselves, stating their name and status (patient, psychiatrist, bike trip enthusiast). In both prevention videos (patient and psychiatrist), narrators followed a similar structure, defining schizophrenia, providing examples of common symptoms, addressing misconceptions and emphasising the importance of seeking care. The psychiatrist, wearing a white coat, presented topics from a medical and external standpoint, speaking in the third person. The patient with schizophrenia filmed himself outdoors, addressing topics in the first person and emphasising his humanity and empowerment. The control video featured a man recounting his first bike trip to Barcelona, Spain.

Attention checks were used to measure inconsistency in responses and data quality. Participants had to answer simple questions about important aspects of the video after watching, as well as to provide their opinions on the video using a visual scale from ‘not at all’ (0) to ‘very much’ (100) for three questions: (1) To what extent did you like this video? (2) To what extent do you think the video is well‐made? (3) To what extent do you find the message of the video interesting?. Concealed within these questions was another attention check: ‘Please place the cursor on the far‐right side (equivalent to 100%)’. The videos were hosted as ‘unlisted’ on YouTube and viewed through the Qualtrics platform.

### 2.3 Measures

The Inventory of Attitudes to Seeking Mental Health Services (IARSSM), a validated French scale adapted from the Mackenzie's Inventory of Attitudes Towards Seeking Mental Health Services (IASMHS) (Mackenzie et al. 2004), was employed to measure attitudes towards seeking mental health services in all groups after video intervention, if applicable (Lheureux 2015). The Inventory consists of 24 items rated on a 0–4 scale, from ‘disagree’ (0) to ‘agree’ (4), and 3 internally consistent factors: psychological openness to seeking help (attitude, items 1, 4, 7, 9, 12, 14, 18, 21), indifference to social stigma (subjective norm, items 3, 6, 11, 16, 17, 20, 23, 24), and the inclination to seek help (behavioural control, items 2, 5, 8, 10, 13, 15, 19, 22). Psychological openness reflects a person's willingness to acknowledge a psychological issue and seek professional care. The inclination to seek help reflects the willingness and perceived ability to seek help for psychological problems. Indifference to stigma refers to the concern an individual would feel if important others discovered they were receiving psychological care. Two attention checks questions were included, one within the IARSSM items instructing participants to mark the option ‘rather agree’ (3), and another asking them to tick ‘none of the sports above’ among demographic questions. The IARSSM total score ranges from 0 to 96, with higher scores reflecting a more positive attitude towards utilising mental healthcare services.

As an exploratory measure, participants' familiarity with the field of mental health assistance was assessed with five yes/no questions before watching the video in all groups, if applicable. Each affirmative response scored one point, resulting in a familiarity score per participant out of 5 (Lheureux 2015). This measure was used to ensure that the groups did not differ in their familiarity with the field of psychology. Indeed, participants' familiarity could influence their responses to the Mental Health Care Attitudes Inventory (Lampropoulos, Fonte, and Apostolidis 2019), potentially biasing our results. The questionnaires were conducted on Qualtrics and took about 20 min to complete.

## 3 Results

We included a total of 147 participants who were then randomly assigned to one of the four conditions. There were 35 participants in the patient video group, 29 in the psychiatrist video group, 45 in the control video group and 38 in the no‐video group. The sample consisted of 104 women, 39 men (26.5%) and 4 individuals identifying as ‘other’ (2.7%). The mean age was 22 years (*M* = 22.4, SD = 1.84, range 18–25). Sociodemographic and IARSSM data are reported in Table 1. Excluded participants were those who did not complete the study up to the last question and those who answered incorrectly to all attention check questions.

**TABLE 1:** Sociodemographic and Inventory of Attitudes to Seeking Mental Health Services (IARSSM) data across groups.

	Patient video group		Psychiatrist video group		Bike video group		No‐video group	
	Mean	SD	Mean	SD	Mean	SD	Mean	SD
Age	22.40	1.85	22.79	1.74	22.18	1.86	22.34	1.89
Familiarity level	2.57	1.14	2.66	0.85	2.70	0.87	3.08	0.88
IARSSM total score	62.43	14.98	67.90	10.33	64.22	11.70	67.95	11.98
Psychological openness to seeking help	22.63	5.42	23.03	5.00	23.13	5.01	23.53	5.20
Indifference to social stigma	20.46	6.50	24.48	4.34	21.76	6.14	23.55	5.30
Inclination to seek help	19.34	6.34	20.38	4.31	19.33	4.58	20.87	5.22

Gender	*N*	%	*N*	%	*N*	%	*N*	%
Femme	25	71.43	21	86.21	30	55.56	28	65.79
Homme	10	28.57	7	34.48	14	22.22	8	26.32
Other	0	0	1	0	1	0	2	0

**FIGURE 1:**
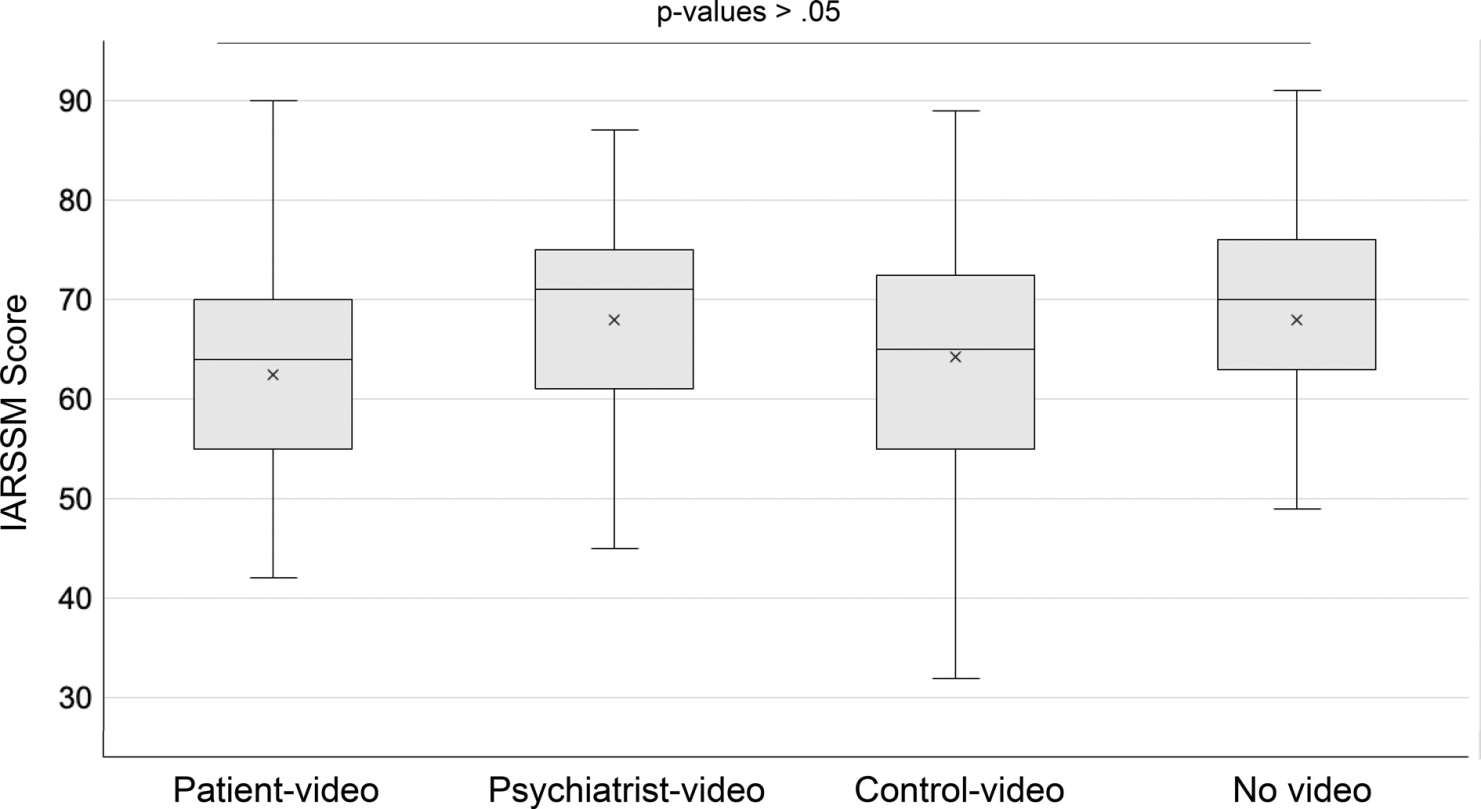
IARSSM (Inventory of Attitudes to Seeking Mental Health Services) total scores across groups.

No significant differences were observed between groups in terms of age (*F* = 0.706, *p* = 0.551), gender (*χ*2_(6)_ = 3.02, *p* = 0.806) and familiarity with mental healthcare scores (*χ*2_(3)_ = 5.74, *p* = 0.125). A Kruskal–Wallis one‐way ANOVA on the total IARSSM score revealed no significant effect of the group on attitude towards mental health care (*χ*2_(3)_ = 6.52, *p* = 0.089, *ε*2 = 0.045). Analysis of the subscores of the IARSSM indicated a nonstatistically significant difference for help‐seeking propensity (*χ*2_(3)_ = 2.84, *p* = 0.417, *ε*2 = 0.019) neither for psychological openness (*χ*2_(3)_ = 0.67, *p* = 0.0878, *ε*2 = 0.005). Conversely, a small but statistically significant effect was found for indifference to stigma (*χ*2_(3)_ = 8.50, *p* = 0.04, *ε*2 = 0.06). Subsequent post hoc pairwise comparisons indicated a trend effect when comparing the patient video group and the psychiatrist video (*p* = 0.07). The patient video group reported (*M* = 20.5, SD = 6.50) slightly lower levels of indifference to stigma compared to the psychiatry video group (*M* = 24.5, SD = 4.35), meaning that the patient video group is more concerned with stigma (Figure 1).

We also tested the association between familiarity (with mental health assistance) score and total IARSSM using a Spearman coefficient. The correlation was not significant (*r*_*s*_ = 0.139, *p* = 0.094). For subscales, no significant associations were found between familiarity and psychological openness (*r*_*s*_ = 0.137, *p* = 0.099), help‐seeking propensity (*r*_*s*_ = 0.132, *p* = 0.113) and indifference to stigma (*r*_*s*_ = 0.060, *p* = 0.471).

## 4 Discussion

The objective of this study was to evaluate the effect of short videos on the attitudes of young people towards seeking mental health care for early psychosis. Contrary to our hypothesis, the results did not reveal significant differences in attitudes towards seeking care between groups. This suggests that the short videos we provided had no differential effect on attitudes towards mental health help‐seeking, contradicting previous results (Aguirre Velasco et al. 2020; Xu et al. 2018). Nevertheless, in Morgan et al.'s (2018) meta‐analysis, social contact interventions such as patient videos only led to weak effect sizes improvement in stigmatising attitudes and desire for social distance towards individuals with mental illness (Morgan et al. 2018). Similarly to our results, no significant difference between patient‐ and psychiatrist‐conducted video intervention was observed across studies (Morgan et al. 2018).

Several factors may have contributed to the lack of difference between video interventions and control conditions. First, numerous mental health studies on the effectiveness of preventive videos include unrepresentative (health) student samples (Morgan et al. 2018). A strength of our study was to exclude individuals working or studying in mental health and to include over 40% of nonstudent participants. Moreover, most studies included in the meta‐analysis by Morgan et al.'s (2018) only assessed the effect of the intervention on stigma or social distance, while our study evaluated two additional dimensions: openness to mental health help‐seeking and willingness to seek help. Furthermore, studies combining social contact and educational interventions reported small to moderate effect sizes, and purely educational interventions had small effect sizes, ranging from 0.08 to 0.47 (Morgan et al. 2018). It is thus a possibility that our nonsignificant results can be attributed to the inherently small size of the effect itself and to that the IARSSM instrument did not detect an effect of small magnitude. Various studies underline the positive effects of gender similarity between learner and speaker in trust in the speaker and motivational variables (Schrader, Seufert, and Zander 2021; Techakesari et al. 2017). Here, we used only male video speakers, and groups were composed of a majority of women, which may have attenuated the positive effects of preventive videos on the leverage of potential attitude towards help‐seeking. Previous studies have found little relationship between fear of stigma and intentions to seek help (Kursite 2020). This could explain why previous studies have found significant reductions in fear of stigma but not in attitude towards help‐seeking, as in our study. Finally, schizophrenia is a highly stigmatised psychiatric disorder, with stigmatisation often occurring even in its early phases (Corcoran 2016). Therefore, a more intensive and repeated intervention may be necessary to observe a statistically significant effect.

Several limitations should be acknowledged. Various help‐seeking facilitators exist, varying for each individual and potentially elevating care‐seeking attitudes irrespective of the video intervention participants underwent. These facilitators include mental health literacy, positive experiences with mental health services, emotional skills and familiarity with mental illness (Aguirre Velasco et al. 2020; Angermeyer et al. 2017; Xu et al. 2018). The latter was not significantly different across intervention groups. However, familiarity was assessed using only five questions, providing a quantitative score but lacking qualitative insights into individuals' relationships with mental illness, such as knowledge or connections with people living with mental illness. While the potential contribution of participants' familiarity with mental illness on attitude towards seeking mental health care was supported by correlated quantitative scores, other facilitators were not assessed in our study. Another limitation is the absence of incentives, which may have attracted participants with preexisting positive attitudes towards mental health, potentially affecting the study's generalizability. Finally, lack of control over the environment in which participants watched videos and answered the questionnaire poses an additional limitation, as the study, conducted entirely online, could not ensure optimal sustained concentration and social desirability bias limitation. Finally, it is important to consider that all actors in the videos were male, while the majority of participants were female. This gender disparity might have induced a similarity bias, potentially facilitating help‐seeking among male participants. To address this limitation, future studies should employ videos featuring actors of varying genders.

By examining the impact of video interventions, this study expands the existing literature on youth attitudes towards mental health help‐seeking for early psychosis. Notwithstanding the proven effect of preventing intervention in promoting mental health help‐seeking, it remains inconclusive as to whether a patient‐ or a psychiatrist‐conducted video is effective in reducing the stigma associated with early stages of psychosis. Long‐term effectiveness and identification of specific factors optimising attitudes towards mental health help‐seeking warrant further investigation.

## Data Availability

The data that support the findings of this study are available on request from the corresponding author. The data are not publicly available due to privacy or ethical restrictions. This research recieved financial support from the Structure Fédérative de Recherche Santé et Société, University Grenoble Alpes.
